# Progesterone-mediated reversal of mifepristone-induced pregnancy termination in a rat model: an exploratory investigation

**DOI:** 10.1038/s41598-023-38025-9

**Published:** 2023-07-06

**Authors:** Christina Camilleri, Stephen Sammut

**Affiliations:** Department of Psychology, Franciscan University of Steubenville, 1235 University Blvd, Steubenville, OH 43952 USA

**Keywords:** Reproductive biology, Endocrinology

## Abstract

Globally, a substantial proportion of pregnancies end in induced (particularly medication) abortion. However, data also indicates a percentage of women who seek assistance in potentially reversing the medication abortion process. While previous literature has suggested the potential for progesterone-mediated reversal of mifepristone-induced abortion, this process has not been effectively investigated pre-clinically. Our study explored the potential reversal of mifepristone-induced pregnancy termination using progesterone in a rat model, following a clear initiation of pregnancy termination. Female Long–Evans rats were divided into three groups (n = 10–16/group): Pregnant control (M−P−), mifepristone-only/pregnancy termination (M+P−) and mifepristone + progesterone (M+P+). Drug/vehicle administration occurred on day 12 of gestation (first-trimester human equivalent). Rat weight was measured throughout gestation. Uterine blood, collected post-drug/vehicle administration, was analyzed spectrophotometrically to measure blood loss. Additionally, at the end of gestation (day 21), ultrasound was utilized to confirm pregnancy and measure fetal heart rate. Number of gestational sacs, uterine weights and diameters were obtained following tissue collection. Our results indicate that progesterone administration following initiation of mifepristone-induced pregnancy termination (indicated by weight loss and uterine bleeding) reversed the process in 81% of rats in the M+P+ group. Following the initial weight loss, these rats proceeded to gain weight at a similar rate to the M−P− group, in contrast to the continued decrease displayed by the M+P− group (and unsuccessful reversals). Moreover, while uterine blood loss was similar to that of the M+P− group (confirming pregnancy termination initiation), number of gestational sacs, uterine weights, diameters, approximate fetal weights and fetal heart rates were similar to the M−P− group. Thus, our results indicate a clear progesterone-mediated reversal of an initiated mifepristone-induced pregnancy termination in a rat model at first-trimester human equivalent, with resultant fully developed living fetuses at the end of gestation, clearly indicating the necessity for further pre-clinical investigation to assist in better informing the scientific/medical communities of the potential implications in humans.

## Introduction

Globally, a substantial portion of pregnancies end in induced abortion e.g.^1–3^. Relative to surgical abortion, the use and availability of drug-induced (medical) abortion has increased substantially over time^4–7^.

Scientific literature continues to address abortion from a variety of angles, with reports of neutral (e.g.^8–10^), positive (e.g.^9,11–15^) and negative (e.g.^8,13,16–23^) outcomes and implications. Moreover, some women, after initiating the abortion process, seek to reverse the abortion following the administration of mifepristone (RU-486, the first drug in the abortion protocol) and prior to the administration of misoprostol e.g.^24,25^.

From a pharmacological perspective, mifepristone acts as a high-affinity competitive progesterone receptor antagonist e.g.^26–28^, binding with an affinity that is reported to be approximately two times that of progesterone in the human uterus^29^. Mifepristone also acts as a glucocorticoid and androgen antagonist^30^. Administration of mifepristone reduces the receptivity of the endometrium and leads to endometrial breakdown e.g.^31,32^ terminating a pregnancy when one is present. This behavior is in line with the withdrawal of progesterone and the resulting endometrial breakdown in menstruation e.g.^33^.

Physiologically, progesterone is key for sustaining a pregnancy, and a reduction in its levels is key to parturition e.g.^34–38^. While contested in certain literature^39–41^, clinically, progesterone/17 alpha-hydroxyprogesterone caproate is used in various pathologies associated with the maintenance of pregnancy, e.g., miscarriage, preterm birth e.g.^37,42–47^.

In concordance with the physiological, pharmacological, and clinical information described above, previous case reports/series^24,25,48^ have indicated that women who were administered progesterone within approximately 72 h of taking mifepristone (to induce an abortion) reported abortion-reversal rates between approximately 60–70%^24^. Such case series suggest that the reversal of a mifepristone-induced abortion through progesterone administration is effective and safe for both the mother and offspring^24^, although conflicting literature also exists^49,50^.

Moreover, a randomized controlled trial by Creinin and colleagues^51^ sought to investigate the potential reversal of a mifepristone-induced abortion through the administration of progesterone. While the clinical trial was halted for safety concerns, the preliminary results, though too low of a sample size to imply statistical significance, suggest the potential for progesterone to successfully reverse a mifepristone-induced abortion. The authors of the study report that four out of the five pregnant women (80%) who remained in the study (or four out of six (67%) if analyzed according to the way groups were originally assigned, known in the literature as “intention-to-treat”) who were administered progesterone following mifepristone reported fetal cardiac activity 2 weeks post-mifepristone administration. This contrasts to two out of five (40%) (or two out of six (33%) based on intention-to-treat analysis) in the placebo group (i.e., received mifepristone, no progesterone)^51^. Additionally, it is important to note that two of the three women who were transported to the hospital for hemorrhaging were in the placebo group (i.e., did not receive progesterone)^51^.

At the preclinical level, previous research by Yamabe et al.^52^ indicated that the co-administration of mifepristone and progesterone resulted in the prevention of pregnancy termination in a rat model. However, in addition to simultaneously administering mifepristone and progesterone, the study reports the administration of the drugs early in the pregnancy (day 7) around the time that the completion of implantation is documented to take place^53^, and does not provide evidence of live fetuses at the end of gestation, given hysterectomies and oophorectomies were conducted, at the latest, 4 days post-drug administration (i.e., day 11). Thus, while suggesting the potential for progesterone administration to reverse the effects of mifepristone in a pregnant animal, it is important to note that such a methodology (i.e., co-administration of progesterone and mifepristone) differs from the non-simultaneous administration utilized in the human protocol (i.e., the administration of progesterone following mifepristone administration)^24,48^, in addition to not clearly providing evidence of survival of embryos through the end of gestation. While all pre-clinical studies are limited in their capacity to extrapolate directly to the clinical level, and no model can ever perfectly replicate all clinical characteristics of a specific condition, it is necessary to strive to provide the best model that potentially, most accurately, provides such a representation. Thus, additional research was necessary in regard to the progesterone-mediated reversal in order to better replicate, more realistically, the non-simultaneous administration, utilized at the clinical level, of mifepristone and progesterone and investigate the potential for the fetuses to survive to the end of gestation (day 21).

Therefore, considering (1) the absence of sufficient pre-clinical studies investigating the potential for the reversal of the antagonistic effects of mifepristone by progesterone and (2) the competitive nature of the mifepristone-progesterone receptor interaction, our research sought to initiate an investigation into the potential for progesterone-mediated reversal of a mifepristone-induced pregnancy termination utilizing our previously established rat model^54^, where the pregnancy termination process has been unambiguously initiated. Due to the complexity of the progesterone dynamic in both parturition and lactation, as well as the complexity of timing of events resulting from the compressed lifespan of the rat with the resultant impact on physiological processes, the focus of this study was solely to investigate the capacity for progesterone-mediated reversal until just prior to delivery, in order to investigate the viability of such a model, with the intention of further investigation, if successful.

## Materials and methods

### Drugs

Mifepristone was purchased from Cayman Chemical (Ann Arbor, MI, USA). Tween^®^ 80 and sesame oil were purchased from Sigma-Aldrich (St. Louis, MO, USA). Progesterone (Ultra micronized), carboxymethylcellulose sodium (CMC-Na) and sodium hydroxide were purchased from VWR (Philadelphia, PA, USA).

### Subjects

Female Long–Evans rats were bred with male rats of the same stock and raised in-house, avoiding any inbreeding. The original breeder pairs were purchased from Hilltop Lab Animals (Scottdale, PA, USA). All animal protocols were approved by the Franciscan University of Steubenville Institutional Animal Care and Use Committee (Protocol Number: 2020-01) and adhere to the Guide for the Care and Use of Laboratory Animals published by the USPHS. The study is reported in accordance with the ARRIVE guidelines. Rats were positioned in such a way that they could see, hear and smell other animals of the same species, under a 12/12 h light–dark cycle (Lights on: 2.15 a.m.) and controlled temperature and humidity (20–26 °C, 30–70% relative humidity), with ad libitum access to standard laboratory chow (RMH 1800, LabDiet) and water. Animal behaviors were monitored daily as an indicator of their health and well-being^55^.

### Experimental procedure

Rat weight and vaginal impedance measurements of single-housed female rats (*n* = 36) were recorded daily beginning at 11–15 weeks of age. Food was also weighed daily in order to track food consumption. Rats were then bred between the ages of 13 and 18 weeks, with day 0 (D0) being the day of breeding. Signs of mating were recorded at the removal of the male, after approximately 6 h.

Animals were randomly assigned to one of three groups: those who received mifepristone followed by vehicle (M+P−; mifepristone-only/pregnancy termination group; n = 10), those who were administered mifepristone followed by progesterone (M+P+; mifepristone + progesterone group; n = 16), and those who received only vehicle and were allowed to carry their pregnancy to term (D21) (M−P−; pregnant control group; n = 10).

Rats in both the M+P+ and M+P− groups were administered mifepristone (50 mg/kg/3 ml, i.g.) in a 0.5–1 ml volume of a CMC-Na (1%) and Tween^®^ 80 (0.2%) suspension on D12 of gestation (first-trimester human equivalent^53,56,57^), followed by one dose of progesterone (in a 0.5–1 ml volume of sesame oil; 150 mg/kg/3 ml, s.c.) or vehicle (sesame oil, s.c.), respectively, approximately 10–15 min following mifepristone administration (dosing, timing, vehicle and route of administration based on pilot data from our lab; data not shown). Rats in the M−P− group were administered vehicle for both injections (1% CMC-Na/0.2% Tween^®^ 80 suspension or sesame oil).

Uterine bleeding and weight loss were indicative of a successful pregnancy termination (M+P−) as per our previous research^54^, and were also required in the mifepristone + progesterone (M+P+) group in order to ensure that the pregnancy termination process had commenced following mifepristone administration.

### Breeding, pregnancy and fetal heart rate confirmation

Vaginal impedance, measured using a Vaginal-Estrous Cycle-Monitor (MK-11, Stoelting, Wood Dale, IL, USA) was measured daily (~ 3.5 h prior to the start of the dark cycle) to determine estrus^58^ and was only collected until the rats were bred. Estrus is indicated by a peak in impedance which is not present in pregnant rats^58–60^. Weight gain was considered as a sign of pregnancy. Additionally, transabdominal ultrasound imaging was performed on rats in all groups (M+P+, M+P−, M−P−) on D21 of pregnancy in order to confirm the presence or absence of fetuses and cardiac activity. Ultrasound imaging was conducted under isoflurane anesthesia, using the EDAN U50 VET Ultrasound Machine, using a Linear array transducer (L15-7b) (Universal Diagnostic Solutions, Inc., Vista, CA, USA). Rats were deeply anesthetized in the induction chamber using 5% isoflurane in oxygen, followed by 2–3.5% in the rebreather nosecone, using the SomnoSuite^®^ Low Flow Anesthesia System (Kent Scientific Corporation, Torrington, CT, USA). The hind limb compression reflex was tested periodically to confirm proper anesthesia. Both uterine horns were scanned for fetuses and fetal heart rates were recorded from random fetuses within each horn, when present.

### Blood/tissue collection and analysis

Beginning on the day following drug/vehicle administration (D13), rats in the M+P− (pregnancy termination) and M+P+ (mifepristone + progesterone) groups were administered cotton (85–90 mg) vaginally in order to collect blood associated with the pregnancy termination process. Cotton was inserted vaginally following the weighing of the animal and removed approximately 3 h later. This procedure was repeated daily in rats experiencing uterine bleeding (M+P+ and M+P−) at the time of weight measurement, until signs of bleeding ceased. For portal vein blood collection from these groups, and for tissue collection from all groups, on D21 of pregnancy, rats were deeply anesthetized using isoflurane and an abdominal incision was made. The uterine horns were carefully exposed, and both the anterior and posterior ends of the uterus and its vasculature carefully ligated. The portal vein was identified and blood collected (in M+P+ and M+P− groups). The uterus was then carefully removed, weighed and the transverse diameter (representing the medio-lateral diameter of the fetus) measured. Additionally, the number of sacs/live fetuses was also recorded. Some uteri contained evidence of fetal demise with significant resorption. These, however, were not counted due to ambiguity in the visual identification at the time of tissue collection of the exact number of fetuses resorbed. Approximate fetal weight of living fetuses was calculated utilizing the uterine weight divided by the number of living fetuses. Following the tissue collection, rats were euthanized via a bilateral thoracotomy and exsanguination. Cotton and portal vein blood samples were stored at − 20 °C for spectrophotometric analysis.

The collected cotton was analyzed utilizing the alkaline hematin technique. This is a common method used both clinically and in research to measure the quantity of uterine blood collected. The addition of an alkaline solution (sodium hydroxide) to blood leads to the conversion of the hemoglobin present in the blood to alkaline hematin, which can be detected spectrophotometrically^61–63^. Briefly, cotton balls were washed repeatedly with a known volume (5 ml) of 5% sodium hydroxide until the washed solution was colorless. The individual rinses with sodium hydroxide were combined into one tube, and the final volume of sodium hydroxide was recorded (*V*_*2*_). Portal vein blood (20 μl) was combined with 4 ml of 5% sodium hydroxide (*V*_*1*_). For analysis, 2 ml of all solutions (i.e., from portal vein blood and individual cotton samples) were spectrophotometrically analyzed at 546 nm. The final volume of uterine blood was calculated using Eq. (1).

1 $$Final\; volume\; (ml)= \frac{0.02\, ml\; (i.e.,\; portal\; vein\; blood\; volume) \times Abs.\; of\; sample \times {V}_{2}}{Abs.\; of\; portal\; vein\; blood \times {V}_{1}}$$

### Statistical analysis

Data is generally presented as mean (M) and standard error of the mean (SEM) unless otherwise indicated. Data analysis was conducted using SigmaPlot 14.0 (Systat Software, Inc.). A two-way repeated measures ANOVA with one factor repetition (gestational day) was utilized to assess differences across groups in average percentage rat weight relative to D1 across gestation (for all groups), as well as differences in uterine blood volume between groups on D13 to D17 (for M+P+, M+P−). Linear regression was utilized to assess differences in the slope of the linear aspect of the percentage rat weight gain (following the drug/vehicle administration) in the M−P− and M+P+ groups for D16 to D21. Comparison of (1) number of gestational sacs (for M+P+, M−P−), (2) vein blood absorbance (for M+P+, M+P−), (3) number of days of investigator-observed bleeding (for M+P+, M+P−), (4) fetal heart rate (for M+P+, M−P−), (5) approximate fetal weight (for M+P+, M−P−) and (6) uterine diameter (for M+P+, M−P−) were conducted utilizing two-tailed independent *t*-tests. A one-way independent measures ANOVA was used to assess differences in uterine weights for all groups. *Post-hoc* Tukey tests were conducted where applicable. For the purpose of analysis, the original mifepristone + progesterone group was considered as two separate groups: successful reversals (i.e., the rats in which the progesterone administration resulted in living fetuses at the time of ultrasound and tissue collection on D21; n = 13; M+P+) and unsuccessful reversals (i.e., the rats in which the progesterone was unsuccessful in preventing the pregnancy termination; n = 3; M+P+ (U)). Differences were considered significant at *p* < 0.05 for all analyses.

### Ethics approval

All animal protocols were approved by the Franciscan University of Steubenville Institutional Animal Care and Use Committee (Protocol Number: 2020-01) and adhere to the Guide for the Care and Use of Laboratory Animals published by the USPHS.

## Results

**Figure 1:**
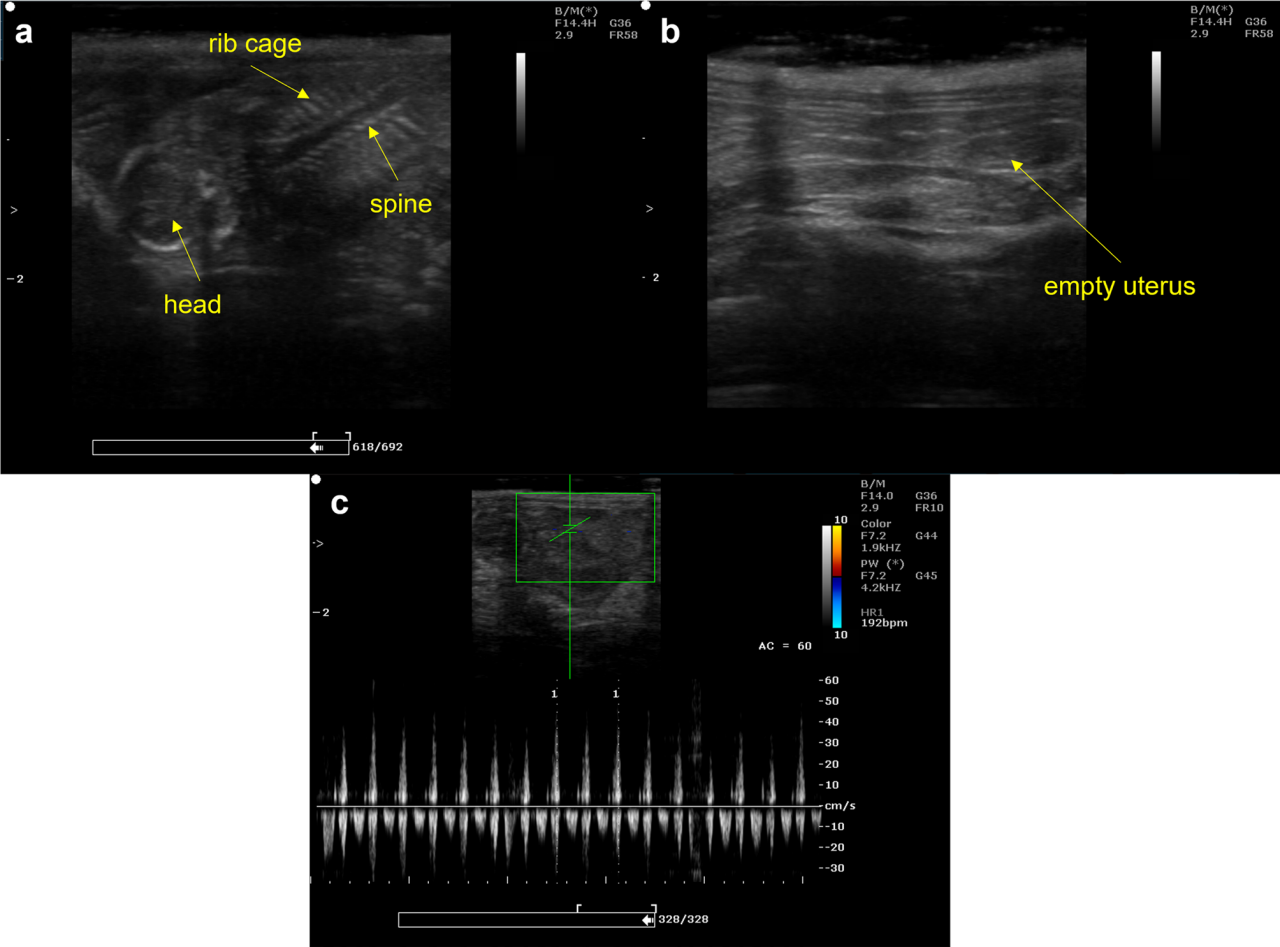
Representative ultrasound images from day 21 of gestation. (**a**) Sample image of a fetus with the head, spine and rib cage labelled. (**b**) Sample image of a non-pregnant uterus. (**c**) Measurement of a fetal heart rate.

Within the groups tested in this study, ultrasound confirmation indicated the presence of live fetuses (Fig. 1a) on D21 in normal pregnant rats (n = 10; M−P−), with the number of fully developed gestational sacs (confirmed at time of tissue collection) ranging from 1 to 13 sacs (M = 9.3, SEM = 1.1). In relation to the reversal group (M+P+), live fetuses were observed in 13 out of 16 (81.3%) rats in this group. Similar to the normal pregnant rats (M−P−), a range of 1–12 fully developed gestational sacs containing living fetuses (n = 13; M = 6.2, SEM = 1.1) was observed in the *successful* reversal rats (M+P+). Analysis of the difference in the number of gestational sacs between the M+P+ and M−P− groups indicated a tendency towards significance (t(21) = 1.96, *p* = 0.063, *r*^2^ = 0.16). No gestational sacs were observed in either the pregnancy termination group (n = 10; M+P−) or the *unsuccessful* reversal group (n = 3, 18.8%; M+P+ (U)) (Fig. 1b).

### Rat weight

**Figure 2:**
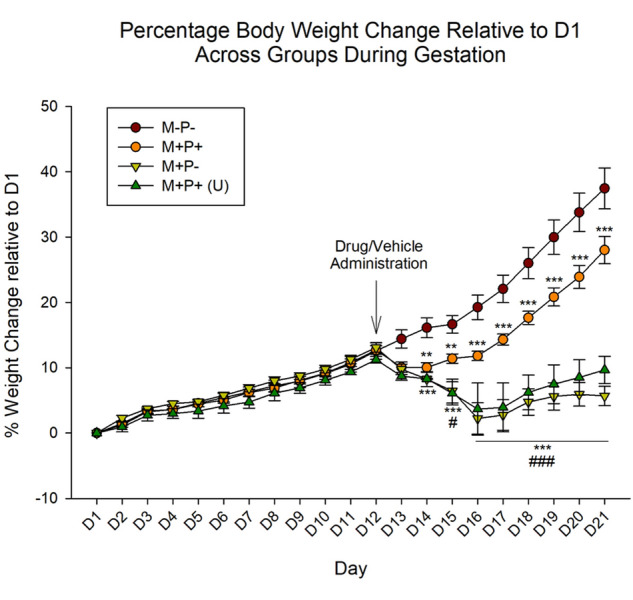
Percentage rat body weight change across gestation period relative to day 1 (D1) of pregnancy. Following drug/vehicle administration, rats in the normal pregnancy group (M−P−; n = 10) continued to show an increase in weight, while rats in the abortion group (M+P−; n = 10) showed significant weight loss, which was also observed in the unsuccessful reversal group (M+P+ (U); n = 3). Rats in the M+P+ group (mifepristone + progesterone / reversal, n = 13) displayed some weight loss followed by a recovery in weight and continued weight gain that paralleled the M−P− group (normal pregnancy). **p < 0.01, ***p < 0.001 (Relative to M−P−); ^#^*p* < 0.05, ^###^*p* < 0.001 (Relative to M+P+). Data is expressed as mean ± SEM. Arrow indicates day of drug/vehicle administration.

Analysis of rat body weight (g) percentage change relative to D1 of gestation (Fig. 2) indicated a significant effect of group (F(3,630) = 13.86, *η*^2^ = 0.12), gestational day (F(20,630) = 86.37, *η*^2^ = 0.25) and the interaction of group and gestational day (F(60,630) = 29.11, *η*^2^ = 0.25) (all *p* < 0.001). *Post-hoc* analysis revealed no significant differences between groups on D1-D13 (all *p* > 0.05, with a tendency towards significance on D13 between both M+P+ (*p* = 0.054) and M+P− (*p* = 0.052) relative to M−P−).

#### Pregnancy termination

Beginning on D14, the average percentage weights of rats in the M+P− group were significantly lower than those in the M−P− group (D14-D21: *p* < 0.001).

#### Reversal

In regard to the reversal group (M+P+), the percentage weight change was significantly lower between days 14 to 21 of gestation (D14-D15: *p* < 0.01; D16-D21: *p* < 0.001) relative to the normal pregnancy group (M−P−), even as the rats in the M+P+ group recommenced gaining weight.

Interestingly, while the percentage weight change of the reversal group was significantly lower as just indicated, there was not a significant difference (F(1,134) = 0.60, *p* > 0.05) in the slope/rate of change in the percentage weight gain between the M−P− and M+P+ groups for D16 to D21.

Additionally, the M+P+ (reversal) group was significantly higher than the M+P− (pregnancy termination) group between D15 to D21 (D15: *p* < 0.05; D16-D21: *p* < 0.001) and the M+P+ (U) (unsuccessful reversal) group between D16 to D21 (D16: *p* < 0.05; D17-D21: *p* < 0.001).

#### Unsuccessful reversal

Rats in the unsuccessful reversal group (M+P+ (U)) were not significantly different across days from the pregnancy termination group (M+P−; all *p* > 0.05). Moreover, the percentage weight change was significantly different on D14 to D21 from the normal pregnancy group (M−P−; D14: *p* < 0.05; D15-D21: *p* < 0.001).

### Uterine bleeding

Given rats in the M−P− (pregnant control) group do not experience bleeding, they were not administered cotton vaginally and therefore were not included in the analysis. Analysis was conducted on blood volumes from cotton that was retrievable vaginally (i.e., not removed by the rat/expelled prior to collection) between days 13 and 17 in the two groups demonstrating bleeding (M+P− and M+P+ groups) (M+P−: *D13*—n = 8, *D14*—n = 6, *D15*—n = 8, *D16*—n = 6, *D17*—n = 5; M+P+: *D13*—n = 9, *D14*—n = 8, *D15*—n = 6, *D16*—n = 2, *D17*—n = 2). While rats that ultimately did not demonstrate a successful reversal (M+P+ (U)) also demonstrated bleeding and analysis of the cotton was conducted where the cotton was retrievable vaginally, this data was not included in the analysis of the spectrophotometric data due to insufficient numbers that would allow for appropriate statistical analysis.

Spectrophotometric analysis of the vein blood absorbance revealed no significant difference between M+P+ and M+P− (t(19) = − 0.70, *p* > 0.05). Additionally, uterine blood volume (measured in ml) analysis for the M+P+ and M+P− groups between D13 and D17 of gestation showed no significant differences between the two groups across days (Group: F(1,27) = 1.11, *η*^2^ = 0.03; Day: F(4,27) = 2.26, *η*^2^ = 0.06; Group × Day: F(4,27) = 1.94, *η*^2^ = 0.05, all *p* > 0.05). Related, analysis of the difference in the number of days of investigator-observed bleeding between the two groups indicated a tendency towards significance (t(20) = 1.76, *p* = 0.094, *r*^2^ = 0.13), with a higher average number of days of bleeding reported in the M+P− group (M = 4.2, SEM = 0.5) relative to the M+P+ group (M = 3.3, SEM = 0.3).

### Ultrasound measurement of fetal heart rates

Analysis indicated no significant difference (t(54) = −1.09, *p* > 0.05, *r*^2^ = 0.02) between the heart rates obtained via ultrasound of fetuses (Fig. 1c) from the M+P+ (reversal group; n = 31 heart rate measurements in beats per minute; M = 221.5, SEM = 6.5) and M−P− (normal pregnancy; n = 25 heart rate measurements in beats per minute; M = 212.0, SEM = 5.3) groups.

### Uterine weight

**Figure 3:**
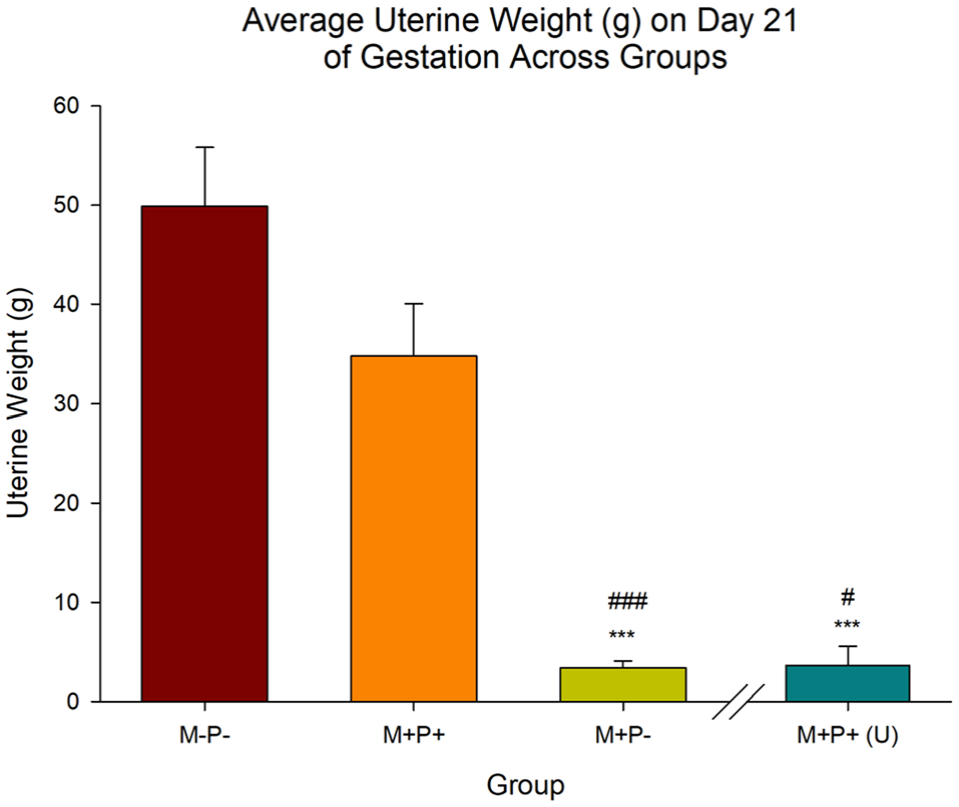
Average uterine weight (g) on day 21 of gestation across groups. Average rat uterine weights were significantly lower in the M+P− (abortion, n = 10) and M+P+ (U) (unsuccessful reversal, n = 3) groups than both M−P− (normal pregnancy, n = 10) and M+P+ (mifepristone + progesterone / reversal, n = 13) groups. ***p < 0.001 (Relative to M−P−); ^#^*p* < 0.05, ^###^*p* < 0.001 (Relative to M+P+). Data is expressed as mean ± SEM.

Analysis of uterine weights (Fig. 3) indicated a significant effect of group (F(3,32) = 18.59, *p* < 0.001, *η*^2^ = 0.64). *Post-hoc* analysis indicated that uterine weights (g) in the M−P− group (normal pregnancy; M = 49.9, SEM = 5.9) were significantly higher than both the M+P− (pregnancy termination; M = 3.4, SEM = 0.7) and M+P+ (U) (unsuccessful reversal; M = 3.7, SEM = 1.9) groups, both *p* < 0.001, but not the reversal group (M+P+; M = 34.8, SEM = 5.3; *p* > 0.05). Additionally, the average uterine weight of the M+P+ group was also significantly higher than both the M+P− (*p* < 0.001) and the M+P+ (U) (*p* < 0.05) groups. There was no significant difference between the uterine weights of the M+P+ (U) and the M+P− groups (*p* > 0.05).

### Uterine diameters and approximate fetal weight

Analysis of the transverse diameter (in mm) of the uterus (representing the medio-lateral diameter of the fetus) revealed no significant difference between the M−P− (M = 18.7, SEM = 0.5) and M+P+ (M = 17.3, SEM = 0.8) groups, t(21) = 1.37, *p* > 0.05, *r*^2^ = 0.08. Additionally, the average uterine weight per fetus (g/fetus), indicative of the approximate fetal weight, for the two groups was also not significantly different (M−P−: M = 5.6, SEM = 0.9; M+P+: M = 6.8, SEM = 2.6; t(21) = − 1.44, *p* > 0.05, *r*^2^ = 0.09).

## Discussion

Our results indicate that in a rat model, (1) one dose of mifepristone at first-trimester human equivalent (~ 4–6 weeks in human embryonic development^53,56,57^), induces a complete pregnancy termination and (2) the administration of progesterone (at a human equivalent to approximately 6–9 h^64^) following mifepristone administration, in a pregnant rat, reverses the effects of the mifepristone, resulting in living offspring at the end of gestation (D21) in the majority (81.3%) of rats in the reversal group (i.e., received mifepristone + progesterone).

The survival of the offspring following the administration of mifepristone appears to directly correlate with the progesterone administration given the clear and evident initiation of the pregnancy termination process based on the presence of the same characteristic physical responses of pregnancy termination initiation in the reversal group as the pregnancy termination group (weight loss and uterine bleeding). In fact, there was no significant difference in regard to uterine bleeding between the two groups. However, in regard to body weight, while never reaching the same percentage body weight gain as that of the normal pregnancy group after drug/vehicle administration, potentially due to some fetal demise in some cases, the successful reversal group displayed weight gain at a rate that paralleled the normal pregnancy group until D21 following the weight loss associated with the pregnancy termination initiation. These results, in addition to the approximate fetal weight, as calculated from the uterine weight per fetus, and the average uterine diameters, appear to support the normal development of the surviving fetuses in the reversal group.

This is in contrast to the rats in which progesterone failed to induce a reversal (18.8%), whose weights paralleled those of the pregnancy termination group with no significant differences across days between the two groups through D21. Moreover, the unsuccessful reversal group also displayed an absence of any gestational sacs and living fetuses as evident through ultrasonography, including the absence of any cardiac activity, and confirmed in the weights of the collected uteri, which were not significantly different from those of the pregnancy termination group.

In relation to the reversal group relative to the normal pregnancy group, percentage weight gain progression (rate of weight gain), uterine weight, and number of fetuses were not significantly different from each other (although the number of fetuses indicated a tendency toward significance), again appearing to support the normal development of the surviving fetuses in the reversal group.

Ultrasound scans and heart rate measurement, in our study, also indicated no difference between the normal pregnancy group and the reversal group. While further investigation is warranted and necessary, this may potentially reflect the absence of detrimental consequences following the administration of progesterone after pregnancy termination initiation using mifepristone, at least at the level of cardiac activity. In fact, a case report of an unsuccessful abortion, using mifepristone, in a twin pregnancy, indicated no postnatal abnormalities^65^. Additionally, previous literature has reported no evidence of a significant increase in major malformations in the continuation of pregnancy following mifepristone exposure^66,67^. Moreover, additional literature has indicated that progesterone/17 alpha-hydroxyprogesterone caproate administration during gestation does not appear to negatively impact the health of the offspring resulting from that pregnancy^39,46,68,69^.

The administration and actions of the natural agonist, progesterone, in the presence of the antagonist, mifepristone, appears to be in concordance with the literature and our understanding of the pharmacological functioning of reversible competitive antagonism^70–75^, where sufficient levels of the agonist can override a given concentration of an antagonist. The higher doses of progesterone necessary can, at least in part, be explained by the necessity to overcome the higher affinity of mifepristone to the progesterone receptor^76^. Another factor that may contribute to the mechanisms at play in this process is the metabolic clearance rate which, in rats, following high acute progesterone levels, has been shown to not only lead to an increase in the progesterone levels, but to also reduce its clearance^77^.

### Limitations and future direction

As with every scientific study, there are limitations that need to be considered. While, as addressed previously, some level of fetal demise was observed in the reversal (mifepristone + progesterone) group despite the survival of a significant number of fetuses equivalent to the normal pregnancy group, further investigation could provide additional details regarding fetal demise, such as number of implantations and resorption rate utilizing techniques such as ammonium sulfide staining^78,79^. Thus, a limitation of this study is that the actual individual fetal weights were not directly measured but were addressed based on the indirect measurement obtained from the uterine weights and the number of observed living fetuses. However, despite this limitation, the average uterine weight per fetus appears to indicate approximate fetal weights that are well in line with those previously documented generally for rats^80–82^.

Another aspect requiring consideration in animal models (including rat models) is the potential differences and similarities in gestation between the animal and human pregnancy. In the case of a rat model, similarities to human pregnancy include hemochorial placentation^83,84^, while differences include a divergence in the primary source of progesterone synthesis that takes place in humans during the luteo-placental shift^34,85^. This shift occurs around the 7th week in a human pregnancy^34^, but does not occur in the rat, where the corpus luteum remains the primary source of progesterone^86^. In this regard, while the capacity to accurately indicate the *exact* human-equivalent timing is a potential limitation, based on previous literature^53,56,57^, our study was conducted prior to the time equivalent to the luteo-placental shift in humans, given that the mifepristone and progesterone administration took place at the equivalent of ~ 4–6 human weeks of pregnancy. Additionally, in relation to the timing of progesterone administration relative to mifepristone, as indicated above, and based on previous literature^64^, it appears that the timing used in this study (approximately 10–15 min after mifepristone administration) is equivalent to between approximately 6 and 9 human hours post-mifepristone administration. While times less than 10 min were not tested in this study, it is anticipated that a shorter gap between the administration of the two drugs would result in better outcomes. The fundamental reasoning behind the timing was to avoid co-administration and the necessity to ensure that we observed the physical symptoms (as reported) of the initiation of the pregnancy termination process. This is fundamental to ensure no ambiguity in the interpretation of the results of progesterone’s capacity to reverse the mifepristone-initiated pregnancy termination.

While the findings from our study, as per any pre-clinical study conducted in animal models, cannot be extrapolated directly to the clinical/human level, they provide the possibility of objectively and ethically investigating progesterone-mediated reversal of mifepristone-induced pregnancy termination. Such a limitation needs to be considered in the interpretation of the data. However, despite this limitation, this study provides a novel and more accurate model for progesterone-mediated reversal of mifepristone-induced pregnancy termination than previous research^52^, with a clear initiation of the pregnancy termination, followed by a recovery that leads to full-term gestation. This study does not address the physiological and behavioral aspects following birth in the case of reversal, in either the mother or the offspring, but provides the foundation necessary for additional research, including at the physiological and behavioral levels. Additionally, the study does not address the critical time period in which mifepristone-induced pregnancy termination can be reversed by the administration of progesterone.

Based on the findings of this current work indicated above, in addition to our previous research pertaining to behavioral and physiological consequences resulting from mifepristone-induced pregnancy termination^54^, future research will seek to integrate these findings into addressing, in greater detail, the impact of mifepristone-induced termination and the progesterone-mediated reversal at the behavioral, physiological and ultimately neurological levels.

## Conclusions

To our knowledge, this is the first study, at the pre-clinical level, to explore and report successful reversal of mifepristone-induced pregnancy termination utilizing non-simultaneous, subsequent administration of micronized progesterone, with clear evidence of initiation of pregnancy termination followed by a clear reversal of the termination process, as evident in the resulting living fetuses at the end of gestation. In addition to providing an objective pre-clinical model for additional investigation of the role of progesterone in reversing mifepristone-induced pregnancy termination, the findings also appear to provide experimental evidence that potentially corroborates previous clinical reports and provide support for the clinical utilization of progesterone in such context.

Additionally, these results, at the very least, emphasize the necessity for extensive additional research, including at the pre-clinical level, into the reversal process in order to inform and ensure the best clinical practices possible, informed by the science, and for the benefit of the patient.

## Data Availability

The data generated and analyzed during the current study are available from the corresponding author on reasonable request.

## Abbreviations

CMC-Na: Carboxymethylcellulose sodium
D followed by a number: Day of gestation
M−P−: Pregnant control group
M+P−: Mifepristone-only/pregnancy termination group
M+P+: Mifepristone + progesterone group; successful reversal group
M+P+ (U): Unsuccessful reversal group
